# Interleaved Optical Coherence Tomography: Clinical and Laboratory Biomarkers in Patients with Diabetic Macular Edema

**DOI:** 10.3390/jpm12050765

**Published:** 2022-05-09

**Authors:** Corina-Iuliana Suciu, Vlad-Ioan Suciu, Ancuţa Cuţaş, Simona Delia Nicoară

**Affiliations:** 1 Department of Ophthalmology, “Iuliu Haţieganu” University of Medicine and Pharmacy, 400012 Cluj-Napoca, Romania; scorinamail2019@gmail.com; 2 Department of Neuroscience, “Iuliu Haţieganu” University of Medicine and Pharmacy, 400012 Cluj-Napoca, Romania; vs_sib@yahoo.com; 3 Department of Medical Statistics, “Iuliu Haţieganu” University of Medicine and Pharmacy, 400012 Cluj-Napoca, Romania; cutas.ancuta@yahoo.com; 4 Department of Ophthalmology, Emergency County Hospital, 400006 Cluj-Napoca, Romania

**Keywords:** diabetic macular edema, OCT, diabetes mellitus, biomarker

## Abstract

(1) Background: The global burden of diabetes mellitus (DM) has been estimated to reach 600 million patients worldwide by 2040. Approximately 200 million people will develop diabetic retinopathy within this time frame. Diabetic macular edema (DME) is a severe, vision-threatening complication that can develop at any stage of diabetic retinopathy, and it represents the main cause of vision loss in patients with DM. Its harmful consequences on visual function could be prevented with timely recognition and treatment. (2) Methods: This study assessed the clinical (demographic characteristics, diabetic evolution, and systemic vascular complications); laboratory (glycated hemoglobin, metabolic parameters, capillary oxygen saturation, and renal function); ophthalmologic exam; and spectral-domain optical coherence tomography (SD–OCT) (macular volume, central macular thickness, maximal central thickness, minimal central thickness, foveal thickness, superior inner, inferior inner, nasal inner, temporal inner, inferior outer, superior outer, nasal outer, and temporal outer thicknesses, disruption of the ellipsoid zone, and disruption of the inner retinal layers (DRIL) parameters in three groups of individuals: healthy controls (HC), patients with DME and type 1 DM (T1DM—group A), and patients with DME and type 2 DM (T2DM—group B) to identify novel correlations between them that would open a path to new pathogenetic hypotheses and, implicitly, to the identification of new therapeutic methods, as part of a tailored treatment within the concept of precision medicine. (3) Results: The duration of DM was significantly longer in group A as compared with group B, as were the prevalence of smoking and systemic vascular complications. Capillary oxygen saturation and estimated glomerular filtration rates were significantly lower, and serum creatinine levels were significantly higher in group A as compared to group B. Regarding the OCT findings, DME had a predominantly eccentric pattern, and the right eye was more severely affected in both groups of patients. Significantly higher values were obtained in group B as compared to group A for the following OCT biomarkers: macular volume, central macular thickness, maximal central thickness, minimal central thickness, foveal thickness, superior inner, inferior inner, nasal inner, inferior outer and nasal outer thickness. The disruption of the ellipsoid zone was significantly more prevalent within group A, whereas the overall disruption of the retinal inner layers (DRIL) was identified significantly more frequently in group B. (4) Conclusions: Whereas systemic and laboratory biomarkers were more severely affected in patients with DME and T1DM, the OCT quantitative biomarkers revealed significantly higher values in patients with DME and T2DM.

## 1. Introduction

The global burden of diabetes mellitus (DM) has been estimated to reach 600 million patients worldwide by 2040 [1]. Approximately 200 million will develop diabetic retinopathy (DR) within this time frame, which will have a significant negative impact on healthcare systems and on patients’ quality of life (QoL) [1]. Diabetic macular edema (DME) is a severe, vision-threatening complication that can develop at any stage of DR, regardless of the duration and severity of DM [2,3].

Optical coherence tomography (OCT) technology has been part of patients’ ophthalmological evaluations over the last 25 years and has become a standard of care, influencing the treatment of millions of people every year [4]. Spectral-domain OCT (SD –OCT) is able to identify subtle retinal changes in asymptomatic eyes before any sign of DME is clinically apparent, making it an essential tool in evaluating all patients with DM. Identifying preclinical biomarkers for DME paves the way for early intervention, leading to better patient outcomes and lower costs for healthcare systems [5,6].

Current research focuses on finding new biomarkers for DME and DR and identifying risk factors for disease progression [7,8,9]. Saxena et al. found that OCT biomarkers, such as central macular thickness (CMT) and macular volume (MV), were increased in patients with DME, considering them as factors that disrupt the ellipsoid zone (EZ), and they correlated with serum biomarkers (urea, creatinine) [8]. Lenis described a specific retinal pattern of lesions in DME, observing its eccentric disposition [9].

Our objectives were to look for OCT (retinal thickness measurements, DRIL parameters, retinal layer morphology, and ETDRS macular map), clinical (body mass index, blood pressure, capillary oxygen saturation, vascular complications, disease history, and ophthalmologic evaluation) and laboratory (metabolic evaluation, inflammatory parameters, and renal function) biomarkers in patients with DME in order to identify novel correlations between them that would open a path to new pathogenetic hypotheses and, implicitly, to the identification of new therapeutic methods as part of a tailored treatment within the concept of precision medicine. To the best of our knowledge, most similar studies were carried out on patients with DM, regardless of the presence of DME. We compared healthy controls with patients with DME and type 1 DM (T1DM) (group A) and type 2 DM (T2DM) (group B) and discussed the results by relating them to published data.

## 2. Materials and Methods

This is an observational, prospective study focused on 3 groups of individuals: healthy controls (HC, n = 35), group A (subjects with DME and T1DM, n = 25), and group B (subjects with DME and T2DM, n = 22). The HC group consisted of subjects without any acute or chronic ophthalmologic disease, logMAR BCVA = 0 and normal OCT scans. group A included subjects older than 18 years of age, with T1DM and DME, and group B included patients older than 18 years of age with T2DM and DME. The diagnosis of DME was established according to the following OCT criteria: CMT ≥ 270 µm and MV ≥ 8.59 µm^3^. Patients with other ophthalmologic conditions (open-angle glaucoma, central retinal vein occlusion, optic neuropathy, and age-related macular degeneration) were excluded from the study.

Age stratification was performed in order to obtain comparable results. All subjects were recruited from the Ophthalmology Department of the Emergency County Hospital, Iuliu Haţieganu University of Medicine and Pharmacy, Cluj-Napoca, Romania. All patients signed an informed consent form upon enrollment. This study adheres to the Declaration of Helsinki, and it was approved by the Ethics Committee of the “Iuliu Haţieganu” University of Medicine and Pharmacy, Cluj-Napoca, Romania (347/1.10.2019) and the Emergency County Hospital, Cluj-Napoca, Romania (37067/19.12.2019).

**Figure 1:**
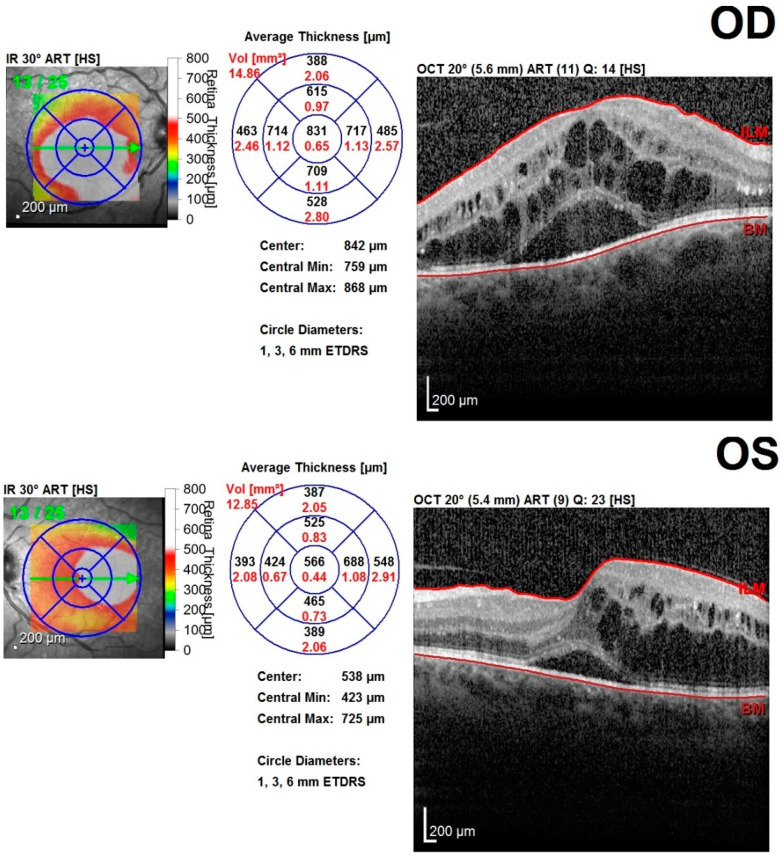

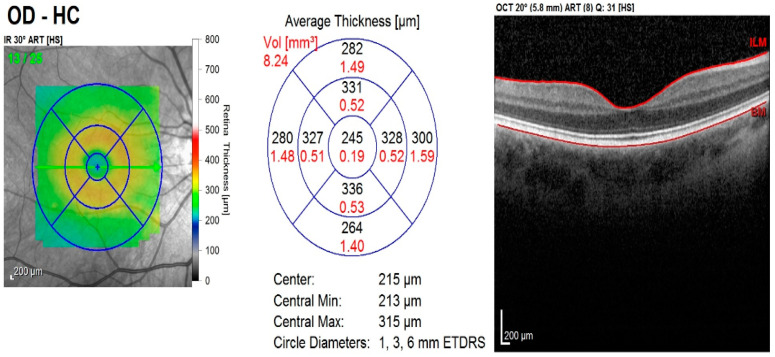
Macular OCT data acquisition from Spectralis Heidelberg SD–OCT device. In this particular case, both eyes have DME with cysts, disruption of the inner retinal layers, small hyperreflective foci, EZ disruption, and detachment of the neuroepithelium. Upper images (OD and OS) belong to the same DME patient with T2DM. The lower image, “OD-HC”, represents a macular OCT scan from a healthy subject (normal macular aspect). Abbreviations: OD—oculus dexter; OS—oculus sinister.

Each patient was examined by the same team of ophthalmologists and the following data were recorded: patient history, best corrected visual acuity (logMAR BCVA), aplanotonometric intraocular pressure measurements, anterior and posterior pole slit-lamp examination results, and results from ophthalmoscopic examination on dilated pupils using Tropicamide 1% eye drops. A macular OCT scan was performed (Heidelberg SPECTRALIS^®^ OCT2 Module Infrared Reflectance with fast macular scan and thickness map rendering using 20 × 20° scan with 25 slices/retina at 200 µm and an automatic, real-time value of 9). All scans were processed using Spectralis Software (Heidelberg Engineering GmbH, Heidelberg, Germany) version 6.10.5 for image quality above 20 dB. The macular module included the ETDRS macular map with the analysis of the MV, maximal central thickness (MCT), minimal central thickness (mCT), CMT, foveal thickness (FT), superior inner thickness, inferior inner thickness, nasal inner thickness, temporal inner thickness, superior outer thickness, inferior outer thickness, nasal outer thickness, and temporal outer thickness. The OCT morphology analysis included the evaluation of cysts, hyperreflective foci, disruption of the inner retinal layers and ellipsoid zone, and detachment of the neuroepithelium (Figure 1).

Laboratory tests included complete blood count and biochemistry blood tests (fasting blood glucose, glycated hemoglobin (HbA1c), high-density (HDL) and low-density (LDL) cholesterol, triglycerides (TG), creatinine, urea, C-reactive protein (CRP), erythrocyte sedimentation rate (ESR)), and urine tests (urine sediment, urinary protein, pH, urinary glucose, cell count, and urinary density) performed at the Central Laboratory of the Emergency County Hospital, Cluj-Napoca, with standardized, accredited methods. General clinical examination of each subject included the measurement of blood pressure, oxygen saturation level, heart rate, weight, and height.

For the statistical analysis, SPSS Statistics v. 28.0.1 (SPSS Inc., Chicago, IL, USA) and Microsoft Office (Microsoft, Redmond, WA, USA) 2016 were used. ANOVA, *t*-tests, Mann–Whitney U tests, and Kruskal–Wallis tests for independent samples were applied. A *p* value < 0.05 was set for statistical significance.

## 3. Results

### 3.1. Baseline Clinical and Laboratory Characteristics of the Study Sample

The mean age was 63.14 ± 11.86 years in the HC group, 56.92 ± 13.08 years in group A, and 63.72 ± 7.56 years in group B. The differences regarding the gender distribution and family history of DM were not statistically significant between the two groups. The comparative clinical and laboratory characteristics of patients are summarized in Table 1.

Significant differences were observed between DME patients with T1DM (21.88 ± 10.29 years) and DME patients with T2DM (11.77 ± 10.31 years) in the duration of diabetes (*p* < 0.001), insulin treatment duration (*p* < 0.0001), HbA1c values (*p* = 0.006), smoking habits (*p* = 0.01), and capillary oxygen saturation (*p* = 0.02). (See Table 1.)

With regard to the vascular complications of DM, higher incidences of complications occurred in DME patients with T1DM (ischemic heart disease *p* = 0.01; peripheral neuropathy *p* = 0.04; diabetic nephropathy *p* = 0.03). The eGFR was lower (*p* = 0.024) and serum creatinine (*p* = 0.01) was proportionally higher in this sample. (See Table 1.)

There were no significant differences between group A and B with regard to the body mass index, fasting blood glucose, systolic blood pressure, diastolic blood pressure, mean blood pressure, heart rate frequency, high blood pressure status, stroke history, mean proteinuria, proteinuria/creatinine ratio, urea, urinary density, urinary pH, total cholesterol, LDLC, HDLC, triglycerides, hemoglobin, ESR, and fibrinogen.

### 3.2. OCT Imaging and Ophthalmologic Biomarkers in DME

**Figure 2:**
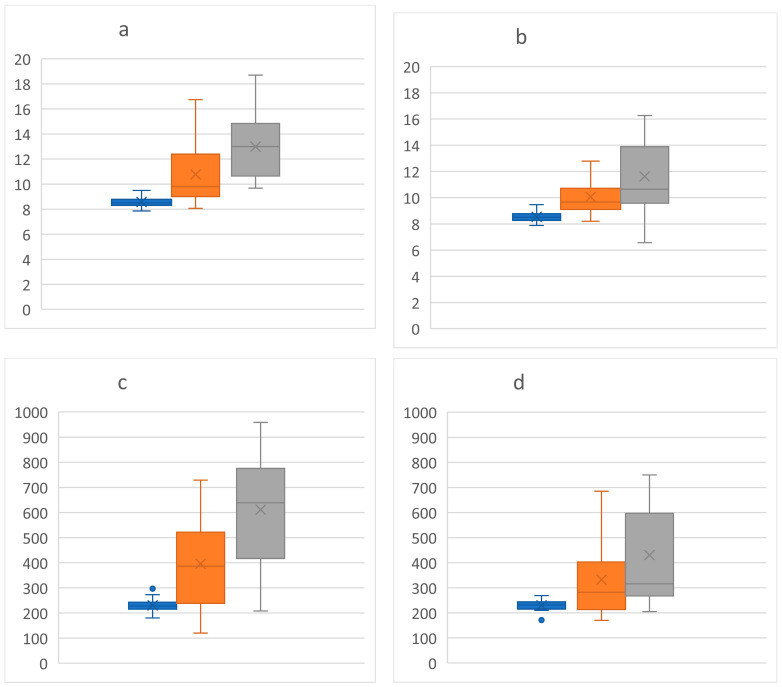
Box plots comparing the macular volume (MV) of the RE (**a**), the LE (**b**), the central macular thickness (CMT) of the RE (**c**) and the LE (**d**) between groups. The measurements are expressed in µm^3^ (y axis) for the MV and µm (y axis) for the CMT. Blue—HC, Orange—group A, and Grey—group B.

When comparing group A with group B, significant differences were obtained in the right eye (RE) with regard to the following parameters: MV, CMT, MCT, mCT, FT, superior inner thickness, inferior inner thickness, nasal inner thickness, inferior outer thickness, and nasal outer thickness. For the left eye (LE), the significance was maintained only for the MV. (See Table 2 and Figure 2).

Regarding the staging of DR, in group A, 56% patients had PDR and 44% had NPDR, and in group B, 55% had PDR and 45% had NPDR. The ellipsoid zone (EZ) disruption was significantly more prevalent in the RE of patients in group A (*p* = 0.05). Overall, the retinal layers were significantly more disrupted in group B (*p* = 0.033). As far as other features were concerned, such as logMAR BCVA, LE EZ disruption, the presence of cysts, hyperreflective foci, and retinal detachment, no significant differences were revealed between groups A and B. (See Table 3).

In our study, an overall disruption of the retinal inner layers (DRIL) was identified in 59% of patients in group B and in 52% of patients in group A (*p* = 0.033). There was a weak, positive correlation between the disruption of the retinal layers and the logMAR BCVA in both groups: r = 0.32 for the RE and r = 0.31 for the LE in group A, and r = 0.38 for the RE and r = 0.43 for the LE in group B.

## 4. Discussion

### 4.1. Clinical and Laboratory Parameters in DME—Risk and Protective Factors

It has been shown that male gender was a risk factor for DME, being more common in that group. Men had higher HbA1c levels than women [7]. Similar to the findings of the authors cited above, we found that the male gender was more prevalent among both DME groups (68% in group A and 59% in group B). Other features associated with DME in our series were: urban environment (>60% in both DME groups), non-academic education (>90% in both DME groups), positive family history of DM (76% in group A and 64% in group B of at least one relative suffering from DM).

The mean duration of DM was also demonstrated to be a risk factor for DME. In a study published by Brazionis et al., the mean duration of DM in patients with DME was 11.7 years [10]. Other authors also confirm this finding, stating that the incidence of DME correlated positively with a longer duration of DM [7,11,12]. In our study, the mean duration of DM in group B was 11.77 ± 10.31 years, similar to the above-mentioned studies [7,11,12], and significantly shorter than in group A (21.88 ± 10.29 years), *p* < 0.0001.

Other noticeable biomarkers associated with the risk for DME were obesity and vascular risk factors. Acan et al. found a mean body mass index (BMI) of 29.25 ± 5.78 in their study sample of DME patients [7], while we found similar results in our study (29.33 ± 5.46 in group A and 28.88 ± 4.36 in group B). High blood pressure was found to be a risk factor for DME, with a difference of +5.1% in the DME group compared to the HC [7]. Within the DME group, 66% of patients had high blood pressure [7], while in our study, a much higher incidence of high blood pressure was found in the DME groups (92% in group A and 86% in group B, *p* = 0.274). According to Endo et al., HbA1c, systolic blood pressure, total cholesterol, and serum triglyceride levels were significantly different between the HC and patients with DME [13].

In a recent study, Klein and colleagues reported the results from 903 subjects with T2DM whom they evaluated for total cholesterol and HDL cholesterol in relation to PDR and DME, concluding that PDR and DME were associated with higher serum levels of total cholesterol [14]. Elevated serum HDL cholesterol has been shown to be a protective factor, while total cholesterol has been shown to be a risk factor for DME in patients without PDR [14]. In our study, the mean HDL cholesterol value was 43.87 ± 14.54 mg/dL for group A and 38.68 ± 8.28 mg/dL for group B, demonstrating that HDL cholesterol cannot be considered a protective factor for DME. Moreover, the mean values between groups had no significant difference [14]. It has been demonstrated that, in patients with DM, LDL cholesterol levels correlated with increased macular thickness [11,12]. LDLC, proteinuria, HbA1c, and age correlated with central subfield macular thickness (CSMT) and MV. According to the same study, HDLC, total cholesterol, and triglyceride levels did not correlate with central subfield macular volume (CSMV) and CSMT [12]. In our study, we did not observe any correlation between blood lipids (HDL, LDL, or triglycerides) and CSMV or CSMT, confirming the results of the above-mentioned study. Both samples showed similar LDLC and HDLC values, with no significant differences. The mean value of HbA1c was significantly higher in group A than in group B (*p* = 0.028), indicating the poorer control of DM in subjects with T1DM.

Chronic kidney disease was shown to be associated with high serum total cholesterol levels and diabetes-related vascular complications [10,14]. In our case series, we found that patients in group B had significantly higher eGFR values (*p* = 0.024), translating into a lower incidence of diabetic nephropathy (*p* = 0.032) than in group A. Serum creatinine levels were also significantly lower in group B as compared to group A (*p* = 0.01) although the differences in proteinuria did not differ significantly between the two groups.

Cholesterol-lowering drugs and high serum HDL cholesterol were related to a lower incidence of DME. In the same study, patients with DME were shown to have HbA1c values greater than 7%, a duration of DM of more than 10–20 years, and higher-than-normal serum creatinine levels. Other risk factors shown to be significantly associated with DME in the aforementioned study were pseudophakia, insulin treatment, and DR severity [7]. Atorvastatin was shown to reduce hard exudates and lipid leakage in the macular region in patients with T2DM [15,16]. Our study evaluated patients with normal LDL cholesterol values, being treated with various statins. However, the time of exposure to statins was difficult to evaluate, and their effect could not be quantified.

With regard to possible pathophysiologic mechanisms, we found significant differences between groups A and B with regard to insulin treatment duration, HbA1c values, smoking habits, capillary oxygen saturation, vascular complications, and renal dysfunction, probably due to the differences between the pathophysiological mechanisms and disease duration. Thus, in T1DM, there is an insufficient or absent secretion of insulin, which is treated solely by insulin substitution, while in T2DM, there is insulin resistance which is treated by different types of drugs [17,18,19]. It is well-known that the pathophysiological events differ in the two types of DM, which translates into different types of tissue damage during the natural course of the disease, and the actions of drugs and their interactions with tissues, as well as cellular signals, are different [17,18].

A significant association between peripheral diabetic neuropathy and DME was found [7]. In our study, all patients (100%) within group A and 86% in group B had peripheral neuropathy.

### 4.2. OCT Imaging Parameters in DME

#### 4.2.1. OCT Morphological Aspects in DME

**Figure 3:**
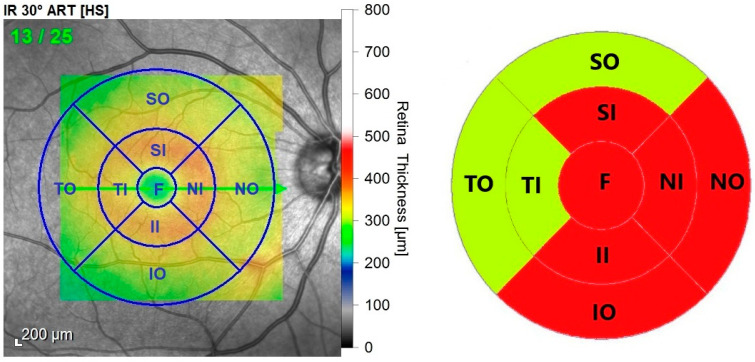
Predominant DME pattern in the retinal sectors of the right eye (images from our study). Abbreviations: F—fovea, SI—superior inner, NI—nasal inner, II—inferior inner, TI—temporal inner, SO—superior outer, NO—nasal outer, IO—inferior outer, TO—temporal outer.

Lenis et al. noted that the morphological aspect of DME is more eccentric, and in most cases, it did not involve all four macular quadrants. The distribution of cysts was predominant in the external (100%) and middle (64.7%) retinal layers, rarely being observed in the inner retinal layers [9]. We found that the predominant pattern of DME in the RE involved the FT, superior, inferior, and nasal inner thicknesses and inferior, nasal outer thickness (µm), involving all the retinal layers, thus confirming the findings published by Lenis et al. (See Figure 3).

A statistically significant correlation between the clinical and OCT patterns of DME and the severity of DR, CMT, and BCVA was proved [20,21].

Cystoid spaces in the retina represent the disruption of the inner blood–retina barrier subsequent to the dysfunction of Müller cells. These specialized cells play an important role in the fluid balance of the retina. When the Müller cells cannot drain the excessive fluid in the macula, or when VEGF disrupts the blood–retina barrier, fluid accumulates and disturbs the normal retinal architecture by creating cysts. Other lesions in DME are the hyper-reflective foci, representing small deposits of lipid and protein within the retina due to leakage of the blood–retina barrier. An increasing number of hyper-reflective foci reveals an evolving DME, being an OCT biomarker for retinal inflammatory reaction [5]. In our research, intraretinal cysts have been identified more frequently in group A than in group B (84% vs. 73%, respectively), as well as hyper-reflective foci (84% vs. 64%, respectively).

The ellipsoid zone (EZ) represents the junction between the inner and outer photoreceptor segments. The disruption of the EZ due to DME leads to poor visual acuity [5]. We have identified EZ disruption more frequently in group A (82%) than in group B (61.5%), but with statistical significance only for the RE (*p* = 0.05).

One observation made by Saxena et al. was that increased serum urea and creatinine were associated with more severe disruption of the external limiting membrane (ELM) and inner segment EZ in patients with T2DM [22]. In a recent paper, Saxena et al. noted that the decrease of logMAR BCVA is associated with the recovery of ELM and EZ [23].

Sharma et al. observed the influence of plasma nitric oxide, lipid peroxide, and reduced glutathione in the structural shaping of the inner segment of the EZ and pigment epithelium [24]. In our case series, the disruption of the EZ in group A was significant (*p* = 0.05) in the RE. The mean duration of DM was longer in group A (21.88 years) versus group B (11.77 years), as well as HbA1c levels (group A 9.47% and group B 8.23%). These observations show that the longer course of DME, as well as poorer control of DM in group A, could explain the higher frequency of EZ disruption compared to that found in the other groups.

Urea and creatinine have been identified as biomarkers for photoreceptor and EZ disruption in DR. Our study confirms this observation: EZ disruption and increased CMT were more frequent in group A, in which creatinine levels were higher as compared to group B (*p* = 0.01). CST was also considered a biomarker for photoreceptor disruption in DME [8].

The accumulation of lipids and proteins in the retina due to the lesions in the blood–retina barrier with non-disrupted ELM determines the thickening of the outer retina. Neuro-sensory detachment appears when the ELM is defective, causing the migration of fluid and proteins into the subretinal spaces [5]. We identified neuro-sensitive retinal detachment caused by subretinal fluid accumulation in 48% of patients in group A and in 45% of patients in group B. Kwon and colleagues found that the subretinal fluid accumulation could be attributed the role of biomarker regarding the severity of DR. However, according to their experience, fluid resorption was not associated with the improvement of the visual function with regard to logMAR BCVA [25].

Shin et al. concluded that the integrity of the junction between the photoreceptors’ inner and outer segments (IS/OS) and of the ELM are biomarkers for assessing the integrity of photoreceptors in DME. They observed that BCVA was influenced by the integrity of IS/OS junction and of the ELM in DME. In the IS/OS junction integrity-disruption group, without loss, the BCVA was significantly superior in comparison to the group with a loss of the IS/OS junction [26,27].

DRIL represents a significant disturbance within the inner retinal layers caused by rupture of bipolar cells’ axons due to excessive mechanical strain in the process of edema [5]. We identified DRIL significantly more frequently in group B as compared to group A. According to Das et al., DRIL is associated with the severity of DR [28]. In our study, the prevalence of PDR was higher, but similar in the two groups, and therefore we cannot support this explanation.

#### 4.2.2. Quantifiable OCT Parameters in DME

Grover et al. [29] studied the CMT on 36 Caucasian individuals with ages ranging from 20 to 84 years and found a mean CMT of 271.4 ± 19.6 µm. Also, Meyer et al. [30] studied the MV in addition to CMT on a similar study group (25 subjects with ages ranging from 69 to 88 years) and observed a CMT of 270.17 µm. In respect to the OCT measurements, for research purposes we chose a cut-off value for CMT of 270 µm and 8.59 µm^3^ for MV because of the similarity between our study sample (Caucasian subjects with similar ages) and the previously cited studies.

According to Saxena et al., CST, cube average thickness, cube volume, and logMAR BCVA demonstrated significant differences between patients with DME and without DME [8]. In the same study, the univariate analysis of DM in relation to the OCT parameters showed proportional relationships and significant results for cube average thickness, central subfield thickness (CST), and central volume.

Endo showed that logMAR BCVA was significantly worse in DME patients as compared to HC [13]. The same study revealed that the CMT was 355 ± 133 µm in the NPDR group and 389 ± 207 µm in the PDR group [13]. In our study, the RE had slightly higher logMAR BCVA in group A (1.08) than in group B (0.94), but without statistical significance. With respect to the LE, both groups had similar logMAR BCVA.

In our study, the MV showed significant differences between groups A and B for both eyes. Regarding the RE, several OCT biomarkers have been significantly modified, such as: CMT, MCT, mCT, FT, superior/inferior/nasal inner thickness, Inferior and Nasal outer thickness. This observation highlights the predominance of DME in the RE in our series. According to Figure 2, the MV (µm^3^) of the RE had higher values than in the LE for groups A and B, being highest in T2DM. This is an interesting finding, given that DM is a systemic metabolic disease that should affect the two eyes symmetrically [31,32,33].

#### 4.2.3. Inter-Ocular Differences in DM

Pekel et al. (2014) published a study on inter-ocular asymmetry regarding the OCT measurements (macular thickness, optic disc, fovea angle values, and foveal visible blood vessel count), taking into account the ocular dominance [34]. They hypothesized that the foveal area should be poorly vascularized in the dominant eye, making the light passage easier. This hypothesis did not prove to be true because the blood vessel count was similar between the two eyes. They concluded that there is no significant symmetry difference between eyes [34]. Our study is limited by the fact that we did not evaluate the eye dominance and therefore cannot assess the relationship between the dominant eye and the more affected eye. However, considering the fact that we found no significant inter-ocular differences within the HC group (RE vs. LE CMV *t*-test *p* = 0.697; and RE vs. LE CMT *t*-test *p* = 0.850), the results of our study show a preference of DME in the right eyes (CMT, MCT, mCT, FT, superior/inferior/nasal inner thickness, inferior and nasal outer thickness). Moreover, previous studies proved that healthy persons display inter-ocular symmetry, while individuals with various eye conditions display asymmetry as the disease progresses [35,36].

Little is known about inter-ocular symmetry and disease predominance or progression. Despite this, inter-ocular asymmetry was proved in other studies although its pathogenic mechanism is currently unclear, requiring further investigation [37,38]. The fluid accumulation in the macula is presumed to be caused by an osmotic disequilibrium with the increase in permeability of the retinal–blood barrier (RBB) and the development of neovascularization [6,37]. The vascular alteration of the tight junctions, loss of endothelial cells and pericytes lead to changes in the trans-endothelial transport [6]. The retinal damage is determined by the lesions in the retinal pigment epithelium, polarization changes and ion/water channel disruption. High serum glucose contributes to aggravating the disequilibrium already in process by the downregulating of claudin5, occludin, and other membrane proteins [6]. Hypoxia activates VEGF and cytokines (TNFα), closing this vicious circle. The disruption of the BRB, intricate subcellular mechanisms (increased oxidative stress by reactive oxygen species, cellular signaling), associated vascular risk factors (atherosclerosis), as well as the clinical course of the disease and other unknown factors could possibly influence the rate of development and severity of DME or DR in one eye more than in the contralateral one [38,39]. On the other hand, studies have shown that anti-VEGF treatment for DME in one eye influenced positively the fellow eye, having good results at follow-up [40].

It has been shown that, in patients with T1DM and strict glycemic control, the incidence of DME decreased by 29% and persisted over long periods of time [41]. Our study evaluated all subjects at presentation, with no data on the evolution of DME over time.

In patients with DME, there is a decrease or absence of reflectivity in the EZ on OCT images. Because the mitochondria are concentrated in the EZ, the visual disturbance in DME is attributed to their dysfunction [23].

Precision diagnosis is the basis of precision medicine, as it reveals the cause of the disorder. With ophthalmology now being more accurate, it relies on cutting-edge investigations which are based on sensitive and specific markers. DME is one of the most severe ocular complications of DM, being a potential cause of vision loss, and it needs to be timely and precisely diagnosed in order to prevent disability. Corroborating the clinical, laboratory, and OCT biomarkers (CMV, CMT, MV, EZ disruption, and DRIL), the clinician is supported in establishing a precise diagnosis that creates the premises for the application of a patient-tailored management.

## 5. Conclusions

The comparative assessment of the two groups of patients with DME showed that the systemic parameters (duration of diabetes, HbA1c, smoking habits, capillary oxygen saturation, eGFR, and creatinine) and the incidence of DM-related complications (ischemic heart disease, peripheral neuropathy, and nephropathy) were significantly more severe in patients with T1DM. Features most commonly associated with DME were male gender, urban environment, non-academic education, positive family history of DM, obesity, and vascular risk factors.

Regarding the OCT data, the right eye was predominantly affected in both DME groups and displayed EZ disruption more frequently. Subjects with T2DM displayed more severe DME patterns consisting in DRIL. The increase in MV (µm^3^) was also more significant in this patient-group. The predominant DME pattern in the RE involved the fovea, superior, inferior, nasal-inner thickness and inferior, nasal-outer thickness. The strengths of our study are that it demonstrates higher values of the CMT and MV in patients with T2DM (group B); it confirms the eccentric pattern of DME; it shows lateralization of the retinal lesions, with the RE being more severely affected; and it outlines the need for a complete evaluation (clinical, laboratory, and ophthalmologic) with close monitoring of all diabetic patients.

Our study is limited by the relatively small sample size and the short evaluation period.

Approaching precision medicine with the help of cutting-edge imaging devices, such as OCT, in correlation with systemic and laboratory biomarkers in order to stratify individual risk and target the complications of DM, aims to maximize treatment strategies (new, targeted molecules) and minimize DM-related disability.

## Figures and Tables

**Table 1:** Characteristics of patients according to the type of DM.

Characteristics	Group A (Patients with DME and T1DM; n = 25; 53%)	Group B (Patients with DME and T2DM; n = 22; 47%)	*p* Value
Clinical characteristics			
Age (years)	56.92 ± 13.08	63.72 ± 7.56	0.016 *
Gender	32% women	41% women	0.268 *
Environment	32% rural	36% rural	0.379 *
Education level	4% academic	9% academic	0.249 *
Duration of hospitalization	1 Day 56%	1 Day 82%	0.076 *
Family history of DM	76% at least one relative	64% at least one relative	0.440 *
BMI (kg/m^2^)	29.33 ± 5.46	28.88 ± 4.36	0.377 *
Insulin treatment	100%	50%	<0.0001 *
Duration of DM (years)	21.88 ± 10.29	11.77 ± 10.31	<0.0001 **
Duration of insulin treatment (years)	21.32 ± 10.35	3.90 ± 5.78	<0.0001 *
Smoking (%)	28%	5%	0.014 *
Capillary O_2_ saturation (%)	96.81 ± 1.56	97.51 ± 0.79	0.028 *
SBP (mmHg)	145.40 ± 21.4	145.68 ± 24.99	0.483 *
DBP (mmHg)	83.24 ± 10.46	85.54 ± 12.14	0.246 *
MBP (mmHg)	103.96 ± 12.43	105.59 ± 15.09	0.345 *
HF (bpm)	73.12 ± 9.05	76.31 ± 10.58	0.140 *
High blood pressure	92%	86%	0.274 *
Stroke history	12%	14%	0.435 *
Ischemic heart disease	84%	55%	0.015 *
Peripheral neuropathy	100%	86%	0.041 *
Diabetic nephropathy	80%	77%	0.032 *
G1	24%	33%	N/A
G2	40%	50%	N/A
G3a	8%	23%	N/A
G3b	4%	0%	N/A
G4	8%	4%	N/A
G5	16%	0%	N/A
Laboratory characteristics			
Normoproteinuria	52%	68%	N/A
Proteinuria 30–300 mg/day	48%	32%	N/A
Proteinuria > 300 mg/day	0%	0%	N/A
Mean proteinuria (mg/day)	40.82 ± 45.02	22.95 ± 38.62	0.079 *
Proteinuria/creatinine ratio	23.08 ± 32.01	51.85 ± 136.40	0.172 *
eGFR (mL/min/1.73 m^2^)	60.77 ± 30.51	77.88 ± 27.02	0.024 *
Creatinine (mg/dL)	1.81 ± 1.68	0.94 ± 0.27	0.010 *
Urea (mg/dL)	64.07 ± 40.03	50.28 ± 20.91	0.078 *
Urinary density	1024.34 ± 4.83	1023.33 ± 4.56	0.239 *
Urinary pH	5.34 ± 0.77	5.02 ± 0.67	0.073 *
Fasting blood glucose (mg/dL)	183.04 ± 97.66	151.90 ± 38.36	0.075 *
HbA1c (%)	9.47 ± 1.9	8.23 ± 1.40	0.028 **
Total cholesterol (mg/dL)	183.5 ± 37.11	173.31 ± 53.89	0.232 *
LDLC (mg/dL)	104.54 ± 28.91	103.59 ± 44.56	0.466 *
HDLC (mg/dL)	43.87 ± 14.54	38.68 ± 8.28	0.070 *
Triglyceride (mg/dL)	180.62 ± 126.46	157.68 ± 78.65	0.235 *
Hemoglobin (g/dL)	13.02 ± 2.21	12.92 ± 1.22	0.419 *
ESR (mm/h)	23.37 ± 18.02	22.5 ± 16.94	0.433 *
CRP (mg/dL)	0.67 ± 0.64	0.40 ± 0.47	0.059 *
Fibrinogen (mg/dL)	391.13 ± 92.90	367.23 ± 81.20	0.184 *

* *t*-test *p* value; ** Kruskal–Wallis test *p* value; Abbreviations: BMI—body mass index; SBP—systolic blood pressure; DBP—diastolic blood pressure; MBP—mean blood pressure; HF—heart rate frequency.

**Table 2:** Comparison of OCT characteristics between HC, group A, and group B.

Characteristics	HC Mean ± SD	Group A Mean ± SD	Group B Mean ± SD	*p* Value between Groups	*p* Value HC vs. Group A	*p* Value HC vs. Group B	*p* Value Group A vs. Group B
RE Macular volume (µm^3^)	8.57 ± 0.4	10.77 ± 2.44	12.99 ± 2.72	<0.0001 **	<0.001 *	<0.001 *	0.011 *, <0.0001 ***
LE Macular volume (µm^3^)	8.53 ± 0.41	10.05 ± 1.49	11.62 ± 2.83	<0.0001 **	<0.001 *	<0.001 *	0.034 *, <0.0001 ***
RE CMT (µm)	230.31 ± 23.04	395.83 ± 186.16	611.64 ± 242.93	<0.0001 **	<0.001 *	<0.001 *	0.005 *, 0.028 ^§^
LE CMT (µm)	229.91 ± 19.59	332.47 ± 147.67	430.73 ± 197.64	<0.0001 **	0.005 *	<0.001 *	0.063 *, 0.13 ^§^
RE Maximal central thickness (µm)	226.02 ± 20.33	347.61 ± 135.43	618.2 ± 206.61	<0.0001 ***	<0.0001 ^§^	<0.0001 ^§^	0.008 *, 0.029 ^§^
LE Maximal central thickness (µm)	322.17 ± 19.83	454.6 ± 137.9	535 ± 170.48	<0.0001 ***	<0.0001 ^§^	<0.0001 ^§^	0.06 *, 0.14 ^§^
RE Minimal central thickness (µm)	226.02 ± 20.33	347.61 ± 135.43	490.75 ± 211	<0.0001 ***	<0.0001 ^§^	<0.0001 ^§^	0.006 *, 0.03 ^§^
LE Minimal central thickness (µm)	225 ± 19.55	321.45 ± 122.72	391.42 ± 169.49	<0.0001 ***	<0.0001 ^§^	<0.0001 ^§^	0.07 *, 0.18 ^§^
RE Foveal thickness (µm)	276.11 ± 21.61	405.95 ± 151.34	559.75 ± 219.36	<0.0001 ***	0.001 ^§^	<0.0001 ^§^	0.006 *, 0.02 ^§^
LE Foveal thickness (µm)	275.97 ± 21.63	385.6 ± 130.16	458.61 ± 169.6	<0.0001 ***	0.001 ^§^	<0.0001 ^§^	0.07 *, 0.16 ^§^
RE Superior inner thickness (µm)	337.8 ± 24.7	441.47 ± 129.9	533.2 ± 158.84	<0.0001 ***	<0.0001 ^§^	<0.0001 ^§^	0.03 *, 0.05 ^§^
LE Superior inner thickness (µm)	341.02 ± 1.9	408.3 ± 91.5	470.23 ± 150.38	0.001 ***	0.001 ^§^	0.004 ^§^	0.07 *, 0.19 ^§^
RE Inferior inner thickness (µm)	338.68 ± 16.88	411.52 ± 92	512.25 ± 146.33	<0.0001 ***	<0.0001 ^§^	<0.0001 ^§^	0.008 *, 0.03 ^§^
LE Inferior inner thickness (µm)	337.26 ± 17.84	411 ± 94.43	459.85 ± 130	<0.0001 ***	0.001 ^§^	<0.0001 ^§^	0.11 *, 0.20 ^§^
RE Nasal inner thickness (µm)	341.65 ± 19.58	423.95 ± 90.16	530.7 ± 137.82	<0.0001 ***	<0.0001 ^§^	<0.0001 ^§^	0.003 *, 0.01 ^§^
LE Nasal inner thickness (µm)	342.79 ± 15.92	409 ± 99.29	468.19 ± 136.06	<0.0001 ***	<0.002 ^§^	<0.0001 ^§^	0.07 *, 0.22 ^§^
RE Temporal inner thickness (µm)	328 ± 17.61	436.52 ± 121.39	536.8 ± 183.53	<0.0001 ***	<0.0001 ^§^	<0.0001 ^§^	0.04 *, 0.10 ^§^
LE Temporal inner thickness (µm)	328.35 ± 15.64	409.95 ± 99.04	474.85 ± 139.56	<0.0001 ***	<0.0001 ^§^	<0.0001 ^§^	0.08 *, 0.13 ^§^
RE Superior outer thickness (µm)	298.6 ± 19.27	387.52 ± 113.16	446.9 ± 131.91	<0.0001 ***	0.001 ^§^	<0.0001 ^§^	0.15 *, 0.08 ^§^
LE Superior outer thickness (µm)	293.91 ± 16.07	361.5 ± 80.68	425.57 ± 140.14	<0.0001 ***	<0.0001 ^§^	<0.0001 ^§^	0.05 *, 0.20 ^§^
RE Inferior outer thickness (µm)	284.91 ± 16.75	349.42 ± 64.33	443.5 ± 161.72	<0.0001 ***	<0.0001 ^§^	<0.0001 ^§^	0.03 *, 0.04 ^§^
LE Inferior outer thickness (µm)	283.11 ± 16.47	362.05 ± 87.2	419.42 ± 128.47	<0.0001 ***	<0.0001 ^§^	<0.0001 ^§^	0.05 *, 0.23 ^§^
RE Nasal outer thickness (µm)	312.85 ± 18.46	376.57 ± 60.52	451.95 ± 126.23	<0.0001 ***	<0.0001 ^§^	<0.0001 ^§^	0.02 *, 0.03 ^§^
LE Nasal outer thickness (µm)	313.11 ± 17.19	373.15 ± 78.03	438.81 ± 123.33	<0.0001 ***	<0.0001 ^§^	<0.0001 ^§^	0.04 *, 0.06 ^§^
RE temporal outer thickness (µm)	282.2 ± 15.3	384.14 ± 93.91	457.5 ± 156.27	<0.0001 ***	<0.0001 ^§^	<0.0001 ^§^	0.13 *, 0.10 ^§^
LE temporal outer thickness (µm)	280.29 ± 14.71	366.9 ± 87.69	420.23 ± 141.61	<0.0001 ***	<0.0001 ^§^	<0.0001 ^§^	0.14 *, 0.28 ^§^

* *t*-test *p* value; ** ANOVA test *p* value; *** Kruskal–Wallis test *p* value; ^§^ Mann–Whitney U test *p* value; Abbreviations: RE—right eye, LE—left eye, CMT—central macular thickness, group A—Patients with macular edema and type I diabetes mellitus, group B—Patients with macular edema and type II diabetes mellitus.

**Table 3:** Ophthalmologic characteristics of the patients according to type of diabetes (with DME).

Characteristics	Group A	Group B	*p* Value
logMAR BCVA—RE	1.08 ± 0.86	0.94 ± 0.51	0.302 *, 0.804 **
logMAR BCV—LE	0.97 ± 0.74	0.98 ± 0.79	0.481 *, 0.686 **
NPDR	44%	45%	0.469 *
PDR	56%	55%	0.469 *
EZ disruption—RE	80%	64%	0.050 *
EZ disruption—LE	84%	59%	0.373 *
DME with cysts	84%	73%	0.249 *
Disorganization of the retinal inner layers	52%	59%	0.033 *
Hyperreflective foci	84%	64%	0.406 *
Retinal detachment	48%	45%	0.144 *

* *t*-test *p* value; ** Kruskal–Wallis test *p* value; Abbreviations: NPDR—non-proliferative diabetic retinopathy; PDR—proliferative diabetic retinopathy; EZ—ellipsoid zone; DME—diabetic macular edema; RE—right eye; LE—left eye.

## Data Availability

Patient data were anonymized. The data are not publicly available due to ethical and privacy restrictions.
